# Prevalence of sexually transmitted infections among foreigners living in Guangzhou, China: a cross-sectional study (2010–2017)

**DOI:** 10.1186/s12879-020-04995-8

**Published:** 2020-05-14

**Authors:** Benard Chimungu, Muqing Fu, Jian Wu, Jiali Wu, Liping Huang, Yingchun Dai, Shixing Tang, Jianming Zhang, Chengsong Wan

**Affiliations:** 1 Department of Microbiology, School of Public Health, Southern Medical University, Guangzhou, 510515 China; 2 Clinical laboratory, Guangdong International Travel Health Care Center, Guangzhou, 510635 China; 3 Department of Epidemiology, School of Public Health, Southern Medical University, Guangzhou, 510515 Guangdong China; 4 Key Laboratory of Tropical Disease Research of Guangdong Province, Southern Medical University, Guangzhou, 510515 China

**Keywords:** HBV, HCV, HIV, *Treponema pallidum*, Prevalence, China

## Abstract

**Background:**

The prevalence of HIV/HCV/HBV/ *Treponema pallidum* is an essential health issue in China. However, there are few studies focused on foreigners living in China. This study aimed to assess the prevalence and socio-demographic distribution of HIV, HBV, HCV, and *T. pallidum* among foreigners in Guangzhou in the period of 2010–2017.

**Methods:**

A cross-sectional study was conducted to screen serological samples of 40,935 foreigners from 2010 to 2017 at the Guangdong International Travel Health Care Center in Guangzhou. Samples were tested for hepatitis B surface antigen (HBsAg), anti-HCV, syphilis antibody (anti-TPPA) and anti-HIV 1 and 2. We collected secondary data from laboratory records and used multiple logistic regression analyses to verify the association between different factors and the seroprevalence of HIV/HBV/HCV/ *T. pallidum*.

**Results:**

The prevalence of HBV/HCV/HIV/ *T. pallidum* was 2.30, 0.42, 0.02, and 0.60%, respectively, and fluctuated slightly for 7 years. The results of multiple logistic regression showed that males were less susceptible to HBV than females (*odds ratio [OR] = 0.77, 95%* CI: 0.67–0.89). Participants under the age of 20 had a lower risk of HBV (OR = 0.25, 95% CI: 0.18–0.35), HCV (OR = 0.06, 95% CI: 0.02–0.18), and *T. pallidum* (OR = 0. 10, 95% CI: 0.05–0.20) than participants over the age of 50. Participants with an education level below high school were more likely to have HBV (OR = 2.98, 95% CI: 1.89–4.70) than others, and businessmen (OR = 3.02, 95% CI: 2.03–4.49), and designers (OR = 3.83, 95% CI: 2.49–5.90) had a higher risk of *T. pallidum* than others. Co-infection involved 58 (4.20%) total cases, and the highest co-infection rate was observed for HBV and *T. pallidum* (2.60%).

**Conclusion:**

The prevalence of HBV/HCV/HIV/ *T. pallidum* was low among foreigners in Guangzhou. Region, gender, age, educational level, and occupation were risk factors for positive infection.

## Background

Sexually transmitted infections (STIs) have been recognized as major public health problems in many countries, especially in developing countries [1]. Hepatitis B virus (HBV) infection is considered to be a serious public health problem worldwide, especially in less developed countries. It is estimated that 70% of new chronic HBV infections occur in low-income countries [2]. More so, The Polaris Observatory’s collaborators reported in a survey of 128 countries that the global average HBV prevalence rate was 4.9%, with China, India, Nigeria, Indonesia, and the Philippines accounting for more than 57% of all HBsAg-positive cases [3]. The major burden from HCV infection comes from chronic infection [4], as 184 million individuals worldwide are chronic carriers of HCV [5, 6]. HIV has been spreading from high-risk populations to the general population [7], and 37 million individuals are living with HIV globally. In addition, around six million individuals are infected with *T. pallidum* [8]. Although *T. pallidum* had been eliminated from China in the 1960s by providing free screening and treatment, the first resurgent cases were recognized in China in 1979, and China’s national surveillance data show a disturbing steady spread of the disease across the country [9]. *T. pallidum* has been found to increase HIV infection by two to five times. HIV infection may also increase the spreading of other sexually transmitted diseases, leading to epidemiological synergies between HIV and other STIs [10]. Thus, awareness of co-infection is important because shared transmission pathways and mechanisms may suggest common preventive interventions. In addition, HBV, HCV, HIV, and syphilis can also be transmitted by mother-to-child or iatrogenic transmission, such as contaminated blood or unsterilized dental needles and syringes.

Guangdong is a province in the south of China with an estimated population of 300,000 foreigners. Guangzhou is the capital city of Guangdong. A population of foreigners lives in Guangzhou mostly for economic reasons. Currently, the prevalence of STIs among this population has not been adequately confirmed. To assess the prevalence of HIV, HBV, HCV, and *T. pallidum* among foreigners living in Guangzhou, we designed a cross-sectional study from 2010 to 2017.

## Methods

### Study design, setting, and subjects

A cross-sectional study was approved by the “Guangdong International Travel Healthcare Center Institutional Review Board Committee.” All foreigners arriving in Guangzhou should attend Guangdong International Travel Health Care for physical examination within 6 months. Except for people with incomplete data (The data is not shown in the text), all the other foreigners were included in our study. This study was conducted anonymously. Within the study period, a total of 40, 935 people participated serological tests, including Antibody test for hepatitis B surface antigen (HBsAg), Antibody test for Hepatitis C Virus (anti HCV), Antibody test for HIV 1 and 2 (anti HIV), and *T. pallidum* gelatin agglutination test (anti *T. pallidum*/TPPA). We collected secondary data for analysis.

### Statistical analysis

The difference in the prevalence of STIs between groups was compared using the χ2 tests. Multiple logistic regression analyses were performed to explore the factors associated with seropositivity. The statistically significant variables, according to the χ2 tests, were included in the multiple logistic regression models to compute the adjusted odds ratios (OR) with 95% confidence intervals (CI). The significance level was set at *P* < 0.05. All of the analyses were performed using SPSS 20.0.

## Results

### Sociodemographic characteristics

Of the 40, 935 participants, 23,309 (56.94%) were male and 17,626 (43.06%) were female. The average ages of the participates were 32.59 ± 11.86 years, with a range of 0–97 years (supplementary Table 1). As shown in Table 1, 45.90% of the participants were undergraduate students (*N* = 18,791), while 72.75% had a college education level or less. The majority of participants were from Europe (31.93%) and North America (22.21%). About 29.56% were students, followed by businessmen (24.08%).

**Table 1:** Demographic characteristics of participants, Guangzhou, 2010–2017

Characteristic	Number %	
Total	40,935	100.00
Exam year		
2010	4089	9.99
2011	4665	11.40
2012	4464	10.91
2013	5287	12.92
2014	5907	14.43
2015	5461	13.34
2016	5605	13.69
2017	5457	13.33
Region		
Africa	5927	14.48
Europe	13,071	31.93
North America	9091	22.21
South America	2269	5.54
Oceania	693	1.69
Asia	9884	24.15
Gender		
male	23,309	56.94
female	17,626	43.06
Age group		
< 20	6492	15.86
20–29	14,236	34.78
30–39	9804	23.95
40–49	5557	13.58
≥ 50	4846	11.84
Education level		
Less than high school	371	0.91
High school	10,620	25.94
Undergraduate	18,791	45.90
Bachelor degree or above	7582	18.52
Unknown	3571	8.72
Occupation		
Business	9856	24.08
Designers/science education	4818	11.77
Students	12,102	29.56
Unemployed	2537	6.20
Others	11,622	28.39
STIs		
HBV	943	2.30
HCV	173	0.42
HIV	7	0.02
TPPA	246	0.60

### Prevalence of STIs

**Fig. 1:**
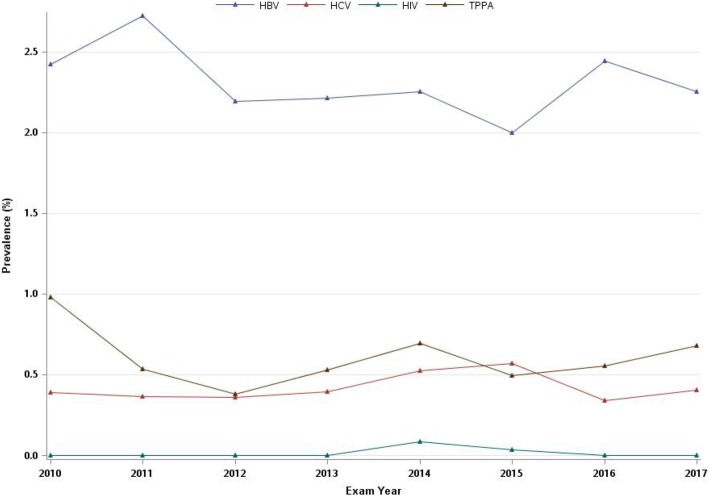
The positive rate of STIs screening during 2010–2017

**Fig. 2:**
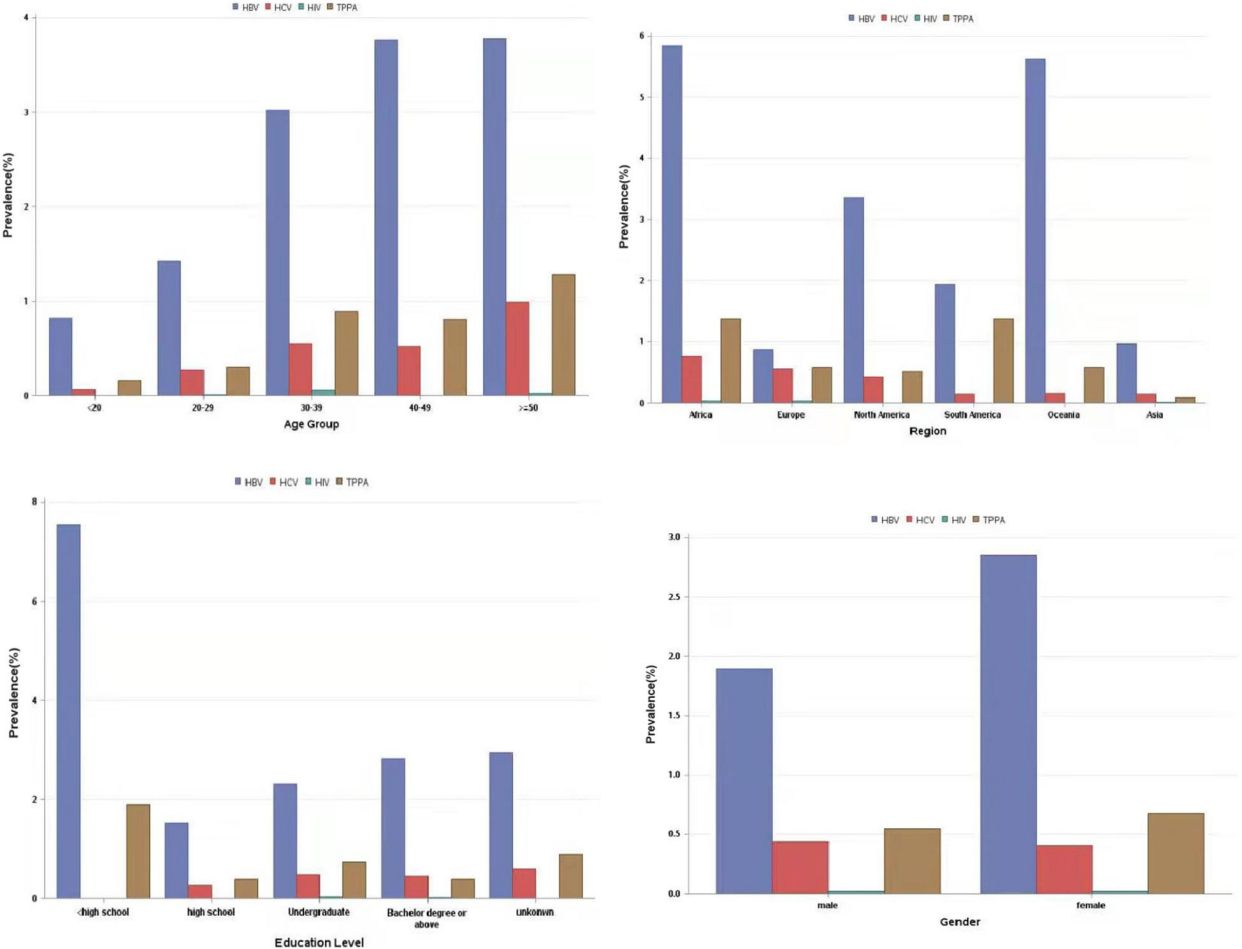
Prevalence of HBV, HCV, HIV and *T. pallidum* by age, region, education level, and gender groups

The prevalence of HBV, HCV, HIV, and *T. pallidum* was 2.30, 0.42, 0.02, and 0.60%, respectively (Table 1), and fluctuated slightly over the 7 years covered by the study (Fig. 1). It was found that 58 (4.2%) cases had multiple infections (Fig. 2), and the highest co-infection rate was observed for HBV and *T. pallidum* (2.6%) (supplementary Table 1).

**Fig. 3:**
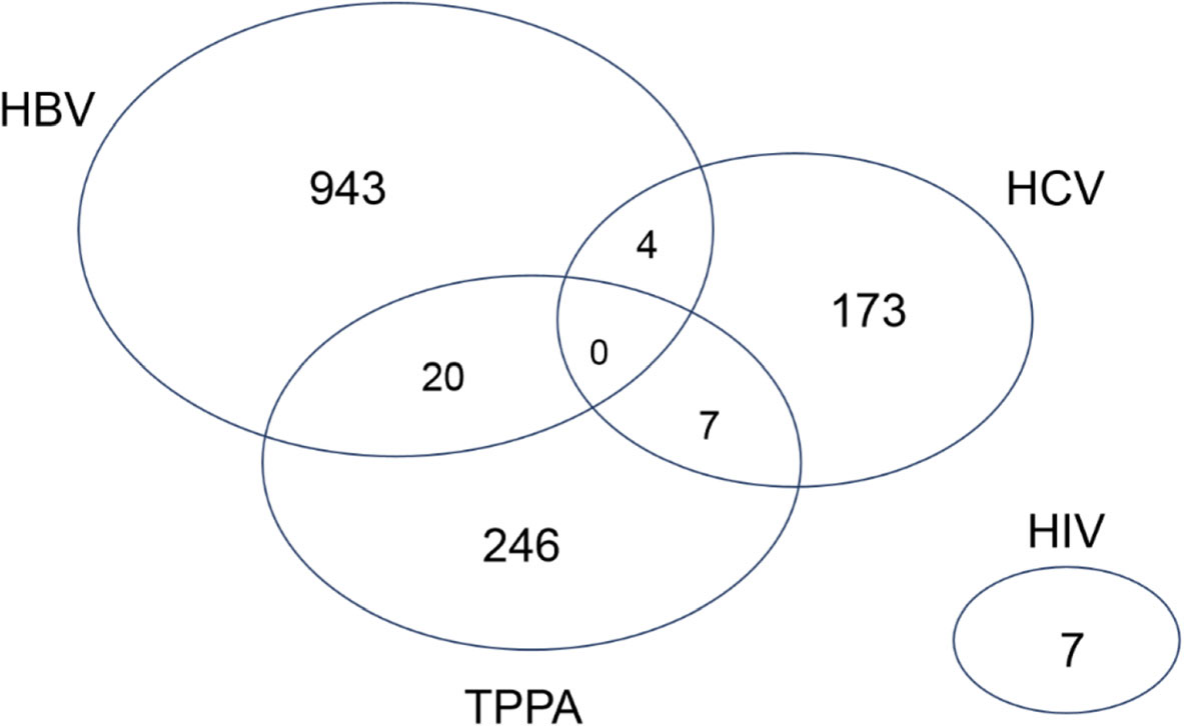
Diagram showing overlap of HBV, HCV, TPPA, and HIV

As shown in Table 2, females had a higher prevalence of HBV (χ2 = 7.58, *P* = 0.01) than males (see Table 2, Fig. 3). There were no differences over the exam year among the STIs. The seroprevalence of HIV, HBV, HCV, and *T. pallidum* presented was different by geographical regions (see Table 2, Fig. 3). There was a significant difference in the seropositivity of HBV between the different age groups (χ2 = 14.15, *P* = 0.01). Educational level differences were also observed in the seroprevalence of HBV (χ2 = 14.94, *P* = 0.01) and *T. pallidum* (χ2 = 14.09, *P* = 0.01). Considering the occupation, there were significant differences for HBV (χ2 = 64.21, *P* < 0.001), HCV(χ2 = 26.19, *P* < 0.001) and *T. pallidum* (χ2 = 155.94, *P* < 0.001). However, perhaps as a consequence of the low number of HIV positive cases, the seropositivity of HIV was not different among the different social demographic characteristics.

**Table 2:** Prevalence of HBV/HCV/HIV/TPPA among individuals with different social demographic characteristics

Characteristic	Total	No. Positive, (%)											
		HBV	χ2	P	HCV	χ2	P	HIV	χ2	P	TPPA	χ2	P
Exam year													
2010	155	99 (2.42)	13.61	0.06	16 (0.39)	8.72	0.27	0 (0)	18.85^a^	0.001^a^	40 (0.98)	11.70	0.11
2011	169	127 (2.72)			17 (0.36)			0 (0)			25 (0.54)		
2012	131	98 (2.20)			16 (0.36)			0 (0)			17 (0.38)		
2013	166	117 (2.21)			21 (0.40)			0 (0)			28 (0.53)		
2014	210	133 (2.25)			31 (0.52)			5 (0.08)			41 (0.69)		
2015	169	109 (2.00)			31 (0.57)			2 (0.04)			27 (0.49)		
2016	187	137 (2.44)			19 (0.34)			0 (0)			31 (0.55)		
2017	182	123 (2.25)			22 (0.40)			0 (0)			37 (0.68)		
Gender													
Male	673	440 (1.89)	7.58	0.01	102 (0.44)	7.61	0.01	4 (0.02)	0.18^a^	0.67^a^	127 (0.54)	0.73	0.39
Female	696	503 (2.85)			71 (0.40)			3 (0.02)			119 (0.68)		
Region													
Africa	474	346 (5.84)	125.37	< 0.001	45 (0.76)	67.12	< 0.001	2 (0.03)	8.91^a^	0.11^a^	81 (1.37)	67.96	< 0.001
Europe	266	114 (0.87)			72 (0.55)			4 (0.03)			76 (0.58)		
North America	389	305 (3.35)			38 (0.42)			0 (0)			46 (0.51)		
South America	78	44 (1.94)			3 (0.13)			0 (0)			31 (1.37)		
Oceania	44	39 (5.63)			1 (0.14)			0 (0)			4 (0.58)		
Asia	118	95 (0.96)			14 (0.14)			1 (0.01)			8 (0.08)		
Age group													
< 20	67	53 (0.82)	14.15	0.01	4 (0.06)	8.02	0.09	0 (0)	6.41^a^	0.17^a^	10 (0.15)	5.94	0.20
20–29	283	202 (1.42)			38 (0.27)			1 (0.01)			42 (0.30)		
30–39	442	296 (3.02)			54 (0.55)			5 (0.05)			87 (0.89)		
40–49	283	209 (3.76)			29 (0.52)			0 (0)			45 (0.81)		
≥ 50	294	183 (3.78)			48 (0.99)			1 (0.02)			62 (1.28)		
Education level													
Less than high school	35	28 (7.55)	14.94	0.01	0 (0)	5.70	0.22	0 (0)	2.81^a^	0.59^a^	7 (1.89)	14.09	0.01
High school	232	161 (1.52)			28 (0.26)			1 (0.01)			42 (0.40)		
Undergraduate	666	435 (2.31)			90 (0.48)			5 (0.03)			136 (0.82)		
Bachelor degree or above	278	214 (2.82)			34 (0.45)			1 (0.01)			29 (0.38)		
Others	158	105 (2.94)			21 (0.59)			0 (0)			32 (0.9)		
Occupation													
Businessmen	227	127 (1.29)	64.21	< 0.001	14 (0.14)	26.19	< 0.001	1 (0.01)	3.51^a^	0.48^a^	85 (0.86)	155.94	< 0.001
Designers	200	105 (2.18)			23 (0.48)			2 (0.04)			70 (1.45)		
Students	340	250 (2.07)			33 (0.27)			3 (0.02)			54 (0.45)		
Unemployed	24	21 (0.83)			1 (0.04)			0 (0)			2 (0.08)		
Others	578	440 (3.79)			102 (0.88)			1 (0.01)			35 (0.3)		

^a^ for likelihood ratio chi-square; No., OR, N/A, and 95% CI represent Number, Odd Rate, No data, and 95% confidence interval, respectively

### Related factors of STIs

The results of multiple logistic regression showed that the seroprevalence of HIV, HBV, HCV, and *T. pallidum* varies according to the geographical region of origin. Infection with HBV, HCV, and *T. pallidum* was the most prevalent in foreigners from Africa. Participants from Africa (OR = 9.13, 95% CI: 6.84–12.19), North America (OR = 2.74, 95% CI: 2.08–3.60), South America (OR = 2.22, 95% CI:1.49–3.30), and Oceania (OR = 6.05, 95% CI: 4.02–9.10) had a higher seroprevalence of HBV than those from Asia. The seroprevalence of HCV in foreigners from Africa (OR = 5.33, 95% CI: 2.88–9.87) and Europe (OR = 3.06, 95% CI: 1.72–5.46) was higher than in those from Asia, and the seroprevalence of *T. pallidum* in Asiatic foreigners was lower than in those from Africa (OR = 17.18, 95% CI: 8.17–36.11) and South America (OR = 19.30, 95% CI: 8.81–42.29).

Among age groups, a significant increase in the positive rate of HBV was observed in the 40–49-year-old participants (OR = 1.05, 95% CI: 0.85–1.30) (see Table 3), and people under 50 had a lower seroprevalence of HCV than people over 50, especially those below 20 (OR = 0.06, 95% CI: 0.02–0.18). The same is true for *T. pallidum* (*P* < 0.001). Educational level differences in seroprevalences were also observed, as people with below high school diplomas had a higher seroprevalence of HBV than other groups (OR = 2.98, 95% CI: 1.89–4.69), and people with bachelor degree had a higher seroprevalence of HBV than other groups (OR = 1.38, 95% CI: 1.07–1.78).

**Table 3:** Association of HBV/HCV with different social demographic characteristics

	HBV			HCV		
Characteristics	No. (%)	OR (95%CI)	*P*-Values	No. (%)	OR (95%CI)	*P*-Values
Exam date			0.34			0.50
2010–2012	324(2.45)	1.07 (0.86,1.33)	0.20	49(0.37)	0.98 (0.58,1.64)	0.68
2014–2017	502(2.23)	0.93 (0.75,1.15)	0.15	103(0.46)	1.12 (0.69,1.82)	0.48
2013	117(2.21)	1.00	N/A	21(0.40)	1.00	N/A
Region			< 0.001			< 0.001
Africa	346 (5.84)	9.13 (6.84,12.19)	< 0.001	45(0.76)	5.33(2.88,9.87)	< 0.001
Europe	114 (0.87)	1.04 (0.77,1.40)	0.79	72(0.55)	3.06 (1.72,5.46)	< 0.001
North America	305 (3.35)	2.74 (2.08,3.60)	< 0.001	38(0.42)	1.53 (0.82,2.86)	0.18
South America	44 (1.94)	2.22 (1.49,3.30)	< 0.001	3(0.13)	0.72 (0.21,2.51)	0.60
Oceania	39 (5.63)	6.05 (4.02,9.10)	< 0.001	1(0.14)	0.56 (0.07,4.27)	0.57
Asia	95 (0.96)	1.00	N/A	14(0.14)	1.00	N/A
Gender			< 0.001			0.18
Male	440(1.89)	0.77 (0.67,0.89)		102(0.44)	1.25 (0.89,1.74)	
Female	503(2.85)	1.00	N/A	71(0.40)	1.00	N/A
Age			< 0.001			< 0.001
< 20	53(0.82)	0.25 (0.18,0.35)	< 0.001	4(0.06)	0.06 (0.02,0.18)	< 0.001
20–29	202(1.42)	0.38 (0.31,0.48)	< 0.001	38(0.27)	0.19 (0.13,0.30)	< 0.001
30–39	296(3.02)	0.76 (0.62,0.94)	0.01	54(0.55)	0.43 (0.29,0.65)	< 0.001
40–49	209(3.76)	1.05 (0.85,1.30)	0.64	29(0.52)	0.48 (0.30,0.76)	0.002
≥ 50	183(3.78)	1.00	N/A	48(0.99)	1.00	N/A
Educational level			< 0.001			0.23
Less than high school	28(7.55)	2.98 (1.89,4.69)	< 0.001	0(0.00)	< 0.00(< 0.00,> 999.)	0.12
High school	161(1.52)	1.39 (1.05,1.84)	0.02	28(0.26)	1.10 (0.58,2.08)	0.18
Undergraduate	435(2.31)	0.83 (0.67,1.04)	0.11	90(0.48)	0.74 (0.45,1.24)	0.17
Bachelor’s degree	214(2.82)	1.38 (1.07,1.78)	0.01	34(0.45)	0.95 (0.52,1.73)	0.54
Others	105(2.94)	1.00	N/A	21(0.59)	1.00	N/A
Occupation			< 0.001			< 0.001
Businessmen	127(1.29)	0.31 (0.25,0.38)	< 0.001	14(0.14)	0.16 (0.09,0.28)	< 0.001
Designers	105(2.18)	0.30 (0.24,0.38)	< 0.001	23(0.48)	0.40 (0.25,0.65)	< 0.001
Students	250(2.07)	0.57 (0.48,0.68)	< 0.001	33(0.27)	0.36 (0.24,0.54)	< 0.001
Unemployed	21(0.83)	0.16 (0.10,0.25)	< 0.001	1(0.04)	0.04 (0.01,0.31)	0.002
Others	440(3.79)	1.00	N/A	102(0.88)	1.00	N/A

No., OR, N/A and 95% CI represent Number, Odd Rate, No data and 95% confidence interval, respectively

For occupation, there were significant differences in HBV for businessmen (OR = 0.31, 95% CI: 0.25–0.38), designers (OR = 0.30, 95% CI: 0.24–0.38), students (OR = 0.57, 95% CI: 0.48–0.68), and unemployed (OR = 0.16, 95% CI: 0.10–0.25) compared to others (Table 3). Notably, *T. pallidum* had a higher prevalence among businessmen (OR = 3.02, 95% CI: 2.03–4.49) and designers (OR = 3.83, 95% CI: 2.49–5.90) than in the other groups (Table 4).

**Table 4:** Associations of HIV/TPPA with different social demographic characteristics

	HIV			TPPA		
Characteristics	No.(%)	OR(95%CI)	*P*-Values	No. (%)	OR(95%CI)	*P*-Values
Exam date			0.99			0.33
2010–2012	0(0.00)	0.96 (< 0.00, > 999.)	0.92	49(0.37)	1.27 (0.82,1.96)	0.24
2014–2017	7(0.03)	> 999. (< 0.00, > 999.)	0.78	103(0.46)	1.12 (0.73,1.71)	0.96
2013	0(0.00)	1.00	N/A	21(0.40)	1.00	N/A
Region			0.50			< 0.001
Africa	2 (0.03)	1.27 (0.05,32.17)	0.34	81(1.37)	17.18(8.17,36.11)	< 0.001
Europe	4 (0.03)	1.03 (0.06,18.35)	0.18	76(0.58)	7.34 (3.53,15.27)	< 0.001
North America	0 (0.00)	< 0.00 (< 0.00,> 999.)	0.14	46(0.51)	5.00 (2.34,10.68)	< 0.001
South America	0 (0.00)	< 0.00 (< 0.00,> 999.)	0.54	31(1.37)	19.30 (8.81,42.29)	< 0.001
Oceania	0 (0.00)	0.00 (< 0.00,> 999.)	0.71	4(0.58)	4.58 (1.36,15.42)	0.01
Asia	1 (0.01)	1.00	N/A	8(0.08)	1.00	N/A
Gender			0.95			0.65
male	4 (0.02)	1.14 (0.21,6.33)		127(0.54)	0.97 (0.73,1.28)	
female	3 (0.02)	1.00	N/A	119(0.68)	1.00	N/A
Age			0.46			< 0.001
< 20	0 (0)	< 0.00 (< 0.00,> 999.)	0.98	10(0.15)	0.10 (0.05,0.20)	< 0.001
20–29	1 (0.01)	0.19 (0.01,3.13)	0.46	42(0.30)	0.16(0.10,0.24)	< 0.001
30–39	5 (0.05)	1.42 (0.16,12.90)	0.39	87(0.89)	0.51 (0.36,0.72)	< 0.001
40–49	0 (0)	< 0.00 (< 0.00,> 999.)	0.98	45(0.81)	0.54 (0.37,0.81)	0.003
≥ 50	1 (0.02)	1.00	N/A	62(1.28)	1.00	N/A
Educational level			0.87			0.12
Less than high school	0 (0.00)	1.41 (< 0.00,> 999.)	0.81	7(1.89)	0.92 (0.38,2.20)	0.30
High school	1 (0.01)	626.90 (< 0.00,> 999.)	0.69	42(0.40)	0.68 (0.41,1.11)	0.78
Undergraduate	5 (0.03)	> 999. (< 0.00,> 999.)	0.29	136(0.82)	0.67 (0.44,1.02)	0.84
Bachelor’s degree	1 (0.01)	409.00 (< 0.00,> 999.)	0.58	29(0.38)	0.48 (0.28,0.83)	0.06
Others	0 (0.00)	1.00	N/A	32(0.90)	1.00	N/A
Occupation			0.39			< 0.001
Businessmen	1 (0.01)	0.98 (0.06,15.98)	0.46	85(0.86)	3.02 (2.03,4.49)	< 0.001
Designers	2 (0.04)	3.05 (0.26,35.20)	0.19	70(1.45)	3.83 (2.49,5.90)	< 0.001
Students	3 (0.02)	3.55 (0.36,35.37)	0.23	54(0.45)	1.98 (1.29,3.06)	0.002
Unemployed	0 (0.00)	0.00 (< 0.00,> 999.)	0.47	2(0.08)	0.32 (0.08,1.34)	0.12
Others	1 (0.01)	1.00	N/A	35(0.30)	1.00	N/A

No., OR, N/A, and 95% CI represent Number, Odd Rate, No data, and 95% confidence interval, respectively

## Discussion

There is an epidemic in China of sexually transmitted diseases and the potential for its continued growth in the future. In addition to sexual transmission, these diseases can also be transmitted through mother-to-child transmission, hospital transmission and so on, so controlling and preventing the spread of STIs are now on the agenda [11, 12]. China set out to expand the comprehensive control program consisting of primary and secondary prevention strategies to ensure that STIs can be prevented and infected individuals can be diagnosed and treated in a timely fashion, especially high-risk individuals [13]. However, available data about the prevalence of STIs in foreigners are limited. This is the first large-scale study that detected the seroprevalences of HBV, HCV, HIV, and *T. pallidum* among foreigners in China.

Of the 40, 935 participants involved, 3.20% (*N* = 1311) had a single infection, and 0.14% (*N* = 58) had multiple infections. A recent study in China showed that the prevalence of HBV in people aged 1–4 years, 5–14 years, and 15–29 years was 0.32, 0.94, and 4.38%, respectively [14], in this research, the seropositivity of HBV was 2.30% (*N* = 943), with the increase of age, the HBV infection rate gradually increased and peaked in the group aged over 50 years, which was in accordance with data for the general population. Foreigners from Africa had the highest proportion of positive HBV rate (5.84%), which is higher than the 4.7% reported in Ethiopia, and lower than the 7.51, 11.2, and 14.96% reported in Benin [15], Cameroon [16], and Burkina Faso [17], respectively.

HCV seroprevalence among foreigners was 0.42%, which is similar to the 0.43% reported in the general population in 2006 in China [18], and it is significantly lower than 2.8%, the average level in the world [19]. Similarly, Africa had the highest rate of HCV infection (0.76%), a value that is higher than the 0.5% reported in Portharcourt [20] and the 0.4% in Ethiopia [21].

Recently, it has been reported that the seroprevalence of *T. pallidum* ranged from 0.31 to 0.70% among blood donors in different areas of China [22–24]. In our study, the seroprevalence of *T.pallidum* (0.60%) was similar in Guangzhou (0.66%) in 2010 [22], and higher than in Nanjing(0.36%) and Xi’an [23]. Africa and South America had the highest rate of *T.pallidum* infection. In sub-Saharan Africa, *T. pallidum* still remains a severe public health problem [25]. When compared with African countries, the seroprevalence of *T. pallidum* infection in our study was significantly lower.

The seroprevalence of HIV in this study was 0.02%. The prevalence rate of HIV infection reported in Guangzhou and Nanjing is 0.02 and 0.08%, respectively [26], whereas in Western China the prevalence of HIV in donors was 0.31% [27, 28]. It is worth noting that there were seven HIV infection cases in total, and five cases were undergraduates, suggesting that college students are still the main group of HIV infection. The prevalence of STIs co-infection was 4.20% in foreigners, and the HBV/ *T. pallidum* co-infection had the maximum proportion. There were no cases involving HIV with any other pathogens. It is possible that the policy related to HIV infection in the country of origin may explain the low prevalence observed in this research. For instance, some travellers may not be allowed to go abroad due to a HIV positive test in their country.

There are several limitations to this study that should be mentioned. First, this article used the secondary data, so the genotypes of various sexually transmitted diseases pathogens were not clear. Second, HIV cases were too small to perform a multiple linear regression, decision trees, or other statistical methods used for analysis [29]. Third, all foreigners who arrive in Guangzhou will accept a physical examination, but some data are incomplete and we removed these data from our study, which may bias the results.

## Conclusion

In conclusion, the epidemiologic data presented in this paper showed the presence of STIs prevalence in foreigners living in Guangzhou. This study showed a low prevalence of STIs among foreigners. Some prevalence were consistent with the local trends. During the survey period, there was no significant decline trend in the prevalence of HBV, HCV, HIV, and *T. pallidum*, so we highlight the need to strengthen the current surveillance program. More observation studies on STIs burden, risk factors, and interventions are needed to provide a solid base for planning and policy change [30, 31]. Furthermore, it is essential to take comprehensive measures including this particular group to control and prevent sexually transmitted infections.

## Supplementary information

**Additional file 1: Table 1.** The prevalence of HBV/HCV/HIV/TPPA detected in samples of 40,935 participants. (XLS 6437 kb)

## Data Availability

The data used and/or analyzed during the current study available from the corresponding author on reasonable request.

## Abbreviations

HIV: Human immunodeficiency virus
HCV: Hepatitis C virus
HBV: Hepatitis B virus
*T. pallidum*: *Treponema pallidum*
HBsAg: Hepatitis B viral surface antigen
Anti-TPPA: Treponemal Pallidum Particle Agglutination
OR: Odds ratio
CI: Confidence interval
STIs: Sexually transmitted infections
